# Maternal diseases and congenital anomalies of the kidney and urinary tract in offspring: a cohort study

**DOI:** 10.1007/s12519-024-00822-1

**Published:** 2024-07-06

**Authors:** Qiang Ma, Ya-Qi Li, Qing-Tang Meng, Bo Yang, Hai-Tao Zhang, Hua Shi, Chang-You Liu, Tian-Chao Xiang, Na Zhao, Jia Rao

**Affiliations:** 1 Department of Nephrology, Tai’an Maternal and Child Health Hospital, Tai’an, China; 2 Department of Nephrology, Children’s Hospital of Fudan University, National Children’s Medical Center, 399 Wanyuan Road, Shanghai, China; 3 Department of Obstetrics, Tai’an Maternal and Child Health Hospital, Tai’an, China; 4 Department of Color Ultrasound, Tai’an Maternal and Child Health Hospital, Tai’an, China; 5 Department of Neonatology, Tai’an Maternal and Child Health Hospital, Tai’an, China; 6 Shanghai Kidney Development and Pediatric Kidney Disease Research Center, Shanghai, China; 7 Shanghai Key Lab of Birth Defect, Children’s Hospital of Fudan University, Shanghai, China; 8 National Key Laboratory of Kidney Diseases, Beijing, China

**Keywords:** Congenital anomalies of the kidney and urinary tract (CAKUT), Kidney anomalies, Predictive model, Risk factors, Urinary tract dilation (UTD)

## Abstract

**Background:**

Congenital anomalies of the kidneys and urinary tract (CAKUT) are the most common cause of prenatally diagnosed developmental malformation. This study aimed to assess the relationship between maternal diseases and CAKUT in offspring.

**Methods:**

This retrospective study enrolled all pregnant women registered from January 2020 to December 2022 at one medical center. Medical information on maternal noncommunicable diseases, including obesity, hypertension, diabetes mellitus, kidney disease, hyperthyroidism, hypothyroidism, psychiatric disease, epilepsy, cancer, and autoimmune disease was collected. Based on the records of ultrasound scanning during the third trimester, the diagnosis was classified as isolated urinary tract dilation (UTD) or kidney anomalies. Multivariate logistic regression was performed to establish models to predict antenatal CAKUT.

**Results:**

Among the 19,656 pregnant women, perinatal ultrasound detected suspicious CAKUT in 114 (5.8/1000) fetuses, comprising 89 cases with isolated UTD and 25 cases with kidney anomalies. The risk of antenatal CAKUT was increased in the fetuses of mothers who experienced gestational diabetes, thyroid dysfunction, neuropsychiatric disease, anemia, ovarian and uterine disorders. A prediction model for isolated UTD was developed utilizing four confounding factors, namely gestational diabetes, gestational hypertension, maternal thyroid dysfunction, and hepatic disease. Similarly, a separate prediction model for kidney anomalies was established based on four distinct confounding factors, namely maternal thyroid dysfunction, gestational diabetes, disorders of ovarian/uterine, and kidney disease.

**Conclusions:**

Isolated UTD and kidney anomalies were associated with different maternal diseases. The results may inform the clinical management of pregnancy and highlight potential differences in the genesis of various subtypes of CAKUT.

**Graphical abstract:**

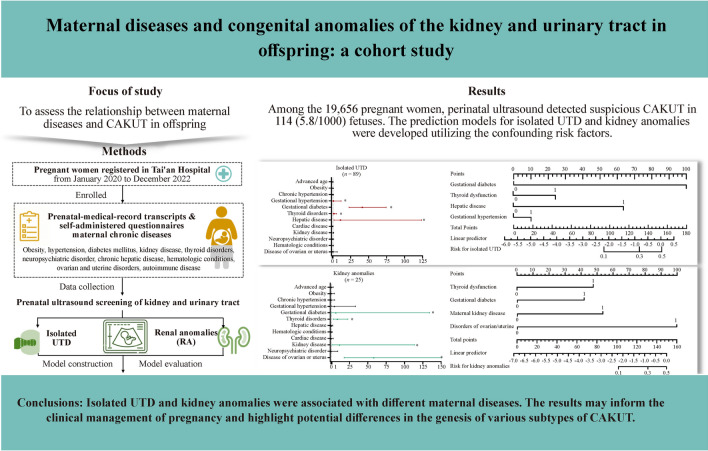

## Introduction

Congenital anomalies of the kidney and urinary tract (CAKUT) are heterogeneous congenital defects in the urinary system that account for 20%–30% of all congenital malformations identified in the prenatal period [1]. CAKUT is a significant cause of morbidity and mortality in neonates. And CAKUT is also the leading cause of end-stage kidney disease in children and adolescents [2]. Early detection and intervention are crucial in managing this condition and improving patient outcomes. Therefore, healthcare professionals must remain vigilant in their efforts to identify and treat CAKUT in a timely and effective manner. Advancements in medical equipment have significantly enhanced the ability to detect kidney and urinary tract anomalies in utero using prenatal ultrasonography. Numerous efforts have been undertaken to screen for CAKUT both during the early prenatal and postnatal periods [3–6]. However, there has been considerable debate surrounding the optimal timing, target population, and cost implications of ultrasound screening for CAKUT.

Much progress has been made in exploring the pathogenesis of CAKUT through various approaches, including genetic and epidemiologic studies. Genetic causes have been identified in only 10%–20% of patients with CAKUT [1, 7, 8]. In contrast, epigenetic studies have explored the environmental effects on gene expression and its contribution to the development of CAKUT. In various epidemiological studies, maternal factors, particularly chronic disease and health conditions during pregnancy, have been recognized as significant contributors among numerous environmental factors [1, 7, 8]. Maternal health conditions may provide more valuable information on high-risk populations who need to conduct screening programs for offspring with CAKUT.

This study investigated the associations between maternal health conditions and CAKUT in offspring through a retrospective birth cohort conducted at the medical center in Tai’an City, China. The specific aim of this study was to explore maternal disease associated with abnormal ultrasound screening results, which could help to establish a population at high risk for CAKUT by perinatal ultrasound screening.

## Methods

### Setting and participants

**Fig. 1:**
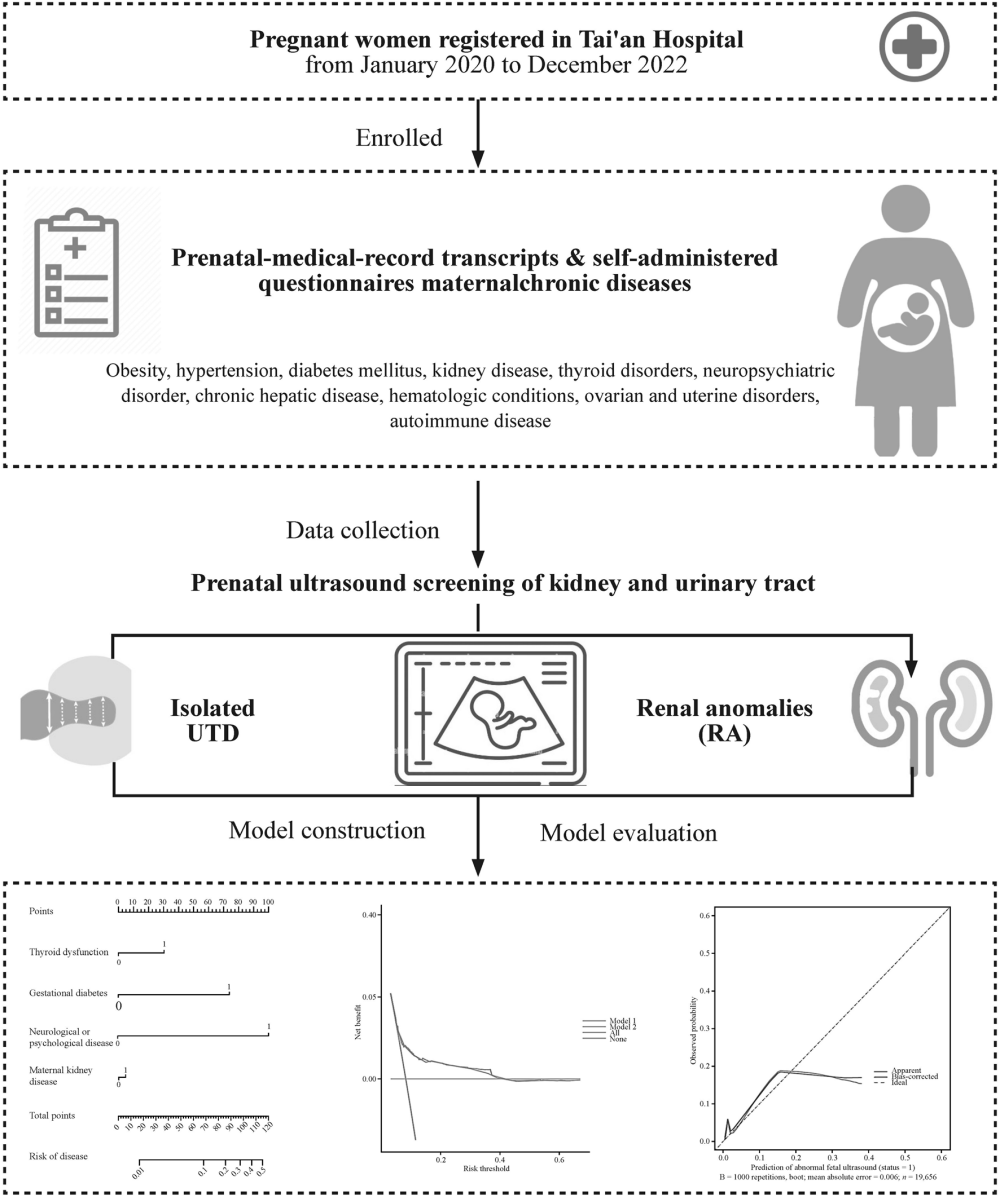
Flowchart of study participants. *CAKUT* congenital anomalies of the kidney and urinary tract, *UTD* urinary tract dilation

This retrospective study consecutively enrolled all pregnant participants from January 2020 to December 2022 in the Tai’an Hospital (Fig. 1), which serves as the primary healthcare provider for the majority of permanent residents and internal migrants in the city. According to the policy and guidelines for pregnancy in China, doctors confirm the pregnancy at the first prenatal check-up and set a plan for the prenatal appointments. At twenty weeks, a scan is booked for each pregnant woman. Data on maternal health conditions was obtained from prenatal medical record transcripts and self-administered questionnaires completed during the first and second/third trimesters of pregnancy. The diagnoses of maternal chronic disease were made at the discretion of the physicians, based on the diagnostic criteria or the definition determined by relevant academic medical societies in China. The study design and data management were approved by the local ethics committee (TA-2019-027), and conducted in accordance with the Helsinki Declaration. Due to the retrospective nature of the study, informed consent was waived. Exclusion criteria included cases missing consent or with incomplete records of pregnancy and prenatal ultrasound information.

### Exposures

The primary exposure factors pertained to medical histories of maternal noncommunicable diseases up to the time of childbirth. A mother was deemed to have a health condition if her diagnosis was reported in the first trimester questionnaire and/or medical records, although the details of the diagnosis were not specified. Maternal chronic diseases included obesity, hypertension, diabetes mellitus, kidney disease, thyroid disorders (hyperthyroidism or hypothyroidism), neuropsychiatric disorders (i.e., psychiatric condition and epilepsy), chronic hepatic disease, hematologic conditions (anemia or thrombocytopenia), ovarian and uterine disorders, and autoimmune disease. The questionnaire administered during the first trimester of pregnancy requested that expectant mothers indicate any medical conditions for which they have received a diagnosis from a physician, excluding obesity, by marking the corresponding checkboxes. The perinatal medical record also documented the presence of certain medical conditions during the index pregnancy, specifically “gestational hypertension” and “gestational diabetes”. Nonetheless, it has been observed that in certain instances, the terms “diabetes” and “gestational diabetes” were simultaneously recorded, likely due to the challenge of distinguishing between them in the absence of blood glucose monitoring before pregnancy. Consequently, these two conditions were combined as the term “diabetes”.

### Outcomes

The primary outcome was CAKUT in the offspring detected by prenatal ultrasound measurements. Ultrasound tests were conducted by ultrasonographers certified by the health administrative department after accumulating more than five years of practice. A GE Voluson 730 scanner (GE Healthcare) with a 3–5 MHz convex-type transducer was used to screen all the participants. The axial planes at the levels of the stomach, kidneys, and bladder were evaluated. The image was recorded only when an abnormality was identified. Urinary tract dilation (UTD) is defined as A1 to A2-3 based on anterior–posterior kidney pelvic diameter measurements and other urinary tract findings [7].

The fetuses of the participants were classified into three groups dependent on ultrasound findings at or after gestational age 28 weeks: (1) “no anomalies” for those without any congenital anomalies; (2) “isolated UTD” for those with UTD without kidney anomalies (KA); and (3) KA for those with anomalies of kidney size, fusion anomalies, kidney ectopia, kidney cysts, and fetal hyperechogenic kidney (Fig. 1).

### Statistical analysis

The proportion of abnormal findings was calculated in the study population. We estimated the risk factors including maternal chronic diseases and disorders during pregnancy. Univariate analysis was initially performed to determine the possible correlation between potential risk factors and abnormal prenatal findings on CAKUT. Associations were estimated by odds ratio (OR) and corresponding 95% confidence intervals (CIs). All significant risk factors (*P* < 0.1) in the univariate logistic regression analysis were eligible for inclusion in the multivariate logistic regression analysis to adjust for confounding factors. Thereafter, we assessed the statistical interaction between the confounding factors. Based on the multivariate logistic regression model, a simplified nomogram was constructed using statistical packages in R. To fit the weights of the models with 5-fold cross-validation, we split the data into five subsets of roughly equal size and interactively used four subsets for interfold training and the fifth subset for interfold testing. Harrell’s concordance index (C-index) was used to quantify the discrimination performance of the simplified nomogram. The performance of the nomogram model was assessed using its calibration which describes the level of agreement between the predicted and actual risks, and is usually evaluated by calibration plot. All statistical analyses were performed using GraphPad Prism 9 (GraphPad Software, La Jolla, California, USA) and statistical packages R version 3.6.1 (R Development Core Team). The packages of rms, Hmisc, pROC, stats, PredictABEL, and rmda were utilized in this process.

## Results

### Baseline characteristics and outcomes

A total of 19,656 pregnant women were incorporated into the study cohort in the medical center of Tai’an City between 2020 and 2022. The mothers enrolled in this study had a median maternal age of 31.2 (interquartile range of 26.1–34.7) years, with 14.1% of mothers classified as advanced maternal age (> 35 years old). Perinatal ultrasound detected suspicious CAKUT in 114 (5.8/1000) fetuses. The most prevalent abnormal findings were isolated UTD (UTD, *n* = 89; unilateral UTD, *n* = 56; bilateral UTD, *n* = 23; unilateral duplex collecting system, *n* = 10), followed by kidney cysts (*n* = 12), horseshoe kidney (*n* = 5), abnormal size or location of kidney (*n* = 3), unilateral kidney agenesis (*n* = 3), and posterior urethral valve with “keyhole” sign (*n* = 2). Among the cases with UTD, there were five fetuses with UTD grade A2-3 and 84 fetuses with grade A1 at 28 weeks’ gestation or later.

### Prognostic factors for antenatally detected kidney malformations

We evaluated the potential risk factors reported in pregnancy charts and the questionnaire between the participant groups with or without abnormal findings through perinatal ultrasound screening (Table 1). The prevalence of noncommunicable illness was relatively high for thyroid dysfunction (2.2%), hepatic disease (1.1%), gestational hypertension (0.8%), gestational diabetes (0.6%), chronic hypertension (0.3%), kidney disease (0.1%), cardiac disease (0.1%), and ovarian and uterine disorders (0.1%). Univariate logistic regression analysis was performed for each potential risk factor. Gestational hypertension, gestational diabetes, thyroid dysfunction, kidney disease, neuropsychiatric disease, anemia, and ovarian and uterine disorders were risk factors for suspicious CAKUT as determined by univariate logistic regression analysis (*P* < 0.05). Thereafter, all these risk factors were entered simultaneously into the multivariate logistic regression analysis to control possible confounding factors. Finally, gestational diabetes, thyroid dysfunction, neuropsychiatric disease, anemia, and ovarian and uterine disorders were proven to be independent risk factors for predicting kidney malformations detected antenatally.

**Table 1:** Univariate and multivariate logistic regression analyses of maternal risk factors associated with kidney and urinary tract anomalies in 19,656 participants screened by antenatal ultrasound scanning

Risk factors	Prenatal ultrasound screening			Univariate analysis		Logistic regression analysis	
	Abnormal (*n* = 114)		Normal (*n* = 19,542)	OR (95% CI)	*P*	β regression coefficient	Standard error
Advanced maternal age							
No		92	16,792	1.0 (reference)			
Yes		22	2750	1.5 (0.9–2.3)	0.110		
Chronic hypertension							
No		114	19.480	1.0 (reference)			
Yes		0	62	1.0 (0.9–1.0)	0.547		
Gestational hypertension						0.5	0.7
No		110	19,382	1.0 (reference)			
Yes		4	160	4.4 (1.6–12.1)	0.002		
Thyroid dysfunction						1.4	0.3
No		101	19,132	1.0 (reference)			
Yes		13	410	6.0 (3.3–10.8)	< 0.001		
Gestational diabetes						3.6	0.3
No		95	19,441	1.0 (reference)			
Yes		19	101	38.5 (22.7–65.4)	< 0.001		
Paternal kidney disease						1.0	1.3
No		113	19,526	1.0 (reference)			
Yes		1	16	10.8 (1.4–82.1)	0.004		
Cardiac disease							
No		114	19,521	1.0 (reference)			
Yes		0	21	1.0 (0.9–1.0)	0.726		
Hepatic disease						2.5	1.2
No		113	19,523	1.0 (reference)			
Yes		1	9	19.2 (2.4–122.9)	< 0.001		
Neurological disease						5.0	1,2
No		113	19,540	1.0 (reference)			
Yes		1	2	86.5 (7.8–960.3)	< 0.001		
Anemia						6.8	0.5
No		92	19,537	1.0 (reference)			
Yes		22	5	934.4 (346.4–2520.7)	< 0.001		
Thrombocytopenia							
No		114	19,537	1.0 (reference)			
Yes		0	5	1.0 (0.9–1.0)	0.864		
High myopia							
No		113	19,542	1.0 (reference)			
Yes		1	0	1.0 (0.7–1.0)	0.016		
HBV infection							
No		112	19,326	1.0 (reference)			
Yes		2	216	1.6 (0.4–6.5)	0.509		
Ovarian cyst						4.7	0.6
No		106	19,531	1.0 (reference)			
Yes		8	11	134.0 (52.8–339.8)	< 0.001		
Uterine diseases						5.5	0.7
No		110	19,537	1.0 (reference)			
Yes		4	5	142.1 (37.7–536.2)	< 0.001		
Syphilis							
No		114	19,519	1.0 (reference)			
Yes		0	23	1.0 (0.9–1.0)	0.714		

*OR* odds ratio, *CI* confidence interval, *HBV* hepatitis B virus

**Fig. 2:**
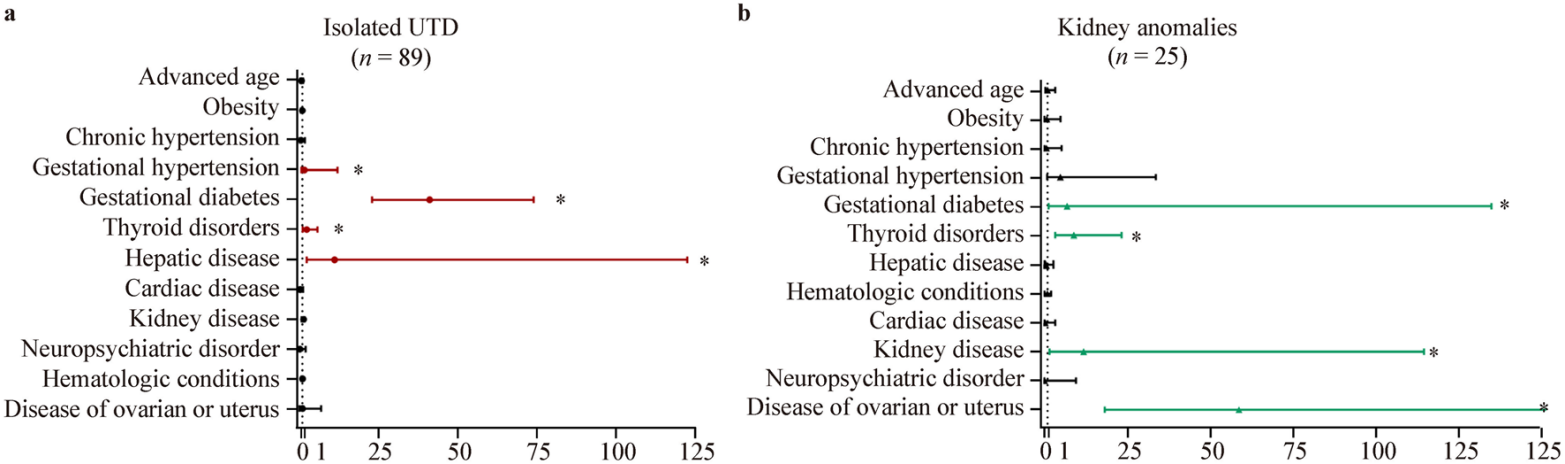
Forest plots showing the adjusted odds ratios for the outcomes versus no defects in association with maternal diseases. The horizontal lines indicate 95% confidence intervals. **a** Isolated urinary tract dilation (UTD) detected antenatally; **b** kidney anomalies detected antenatally. ^*^P < 0.05

Regarding isolated UTD, a higher risk of isolated UTD was observed in the fetuses from mothers with gestational diabetes (adjusted OR = 41.4, 95% CI = 23.1–74.2), gestational hypertension (adjusted OR = 0.69, 95% CI = 0.19–2.5), thyroid dysfunction (adjusted OR = 2.4, 95% CI = 1.1–5.9), and hepatic disease (adjusted OR = 11.2, 95% CI = 0.9–141.6; Fig. 2a). In addition, a higher risk of KA was observed in the fetuses from mothers with thyroid dysfunction (adjusted OR = 8.9, 95% CI = 3.3–23.8), gestational diabetes (adjusted OR = 6.8, 95% CI = 1.3–533.7), ovarian and uterine disorders (adjusted OR = 58.2, 95% CI = 18.1–533.4), and kidney disease (adjusted OR = 11.8, 95% CI = 1.6–134.5; Fig. 2b).

### Development of a nomogram predicting kidney anomalies detected antenatally

**Fig. 3:**
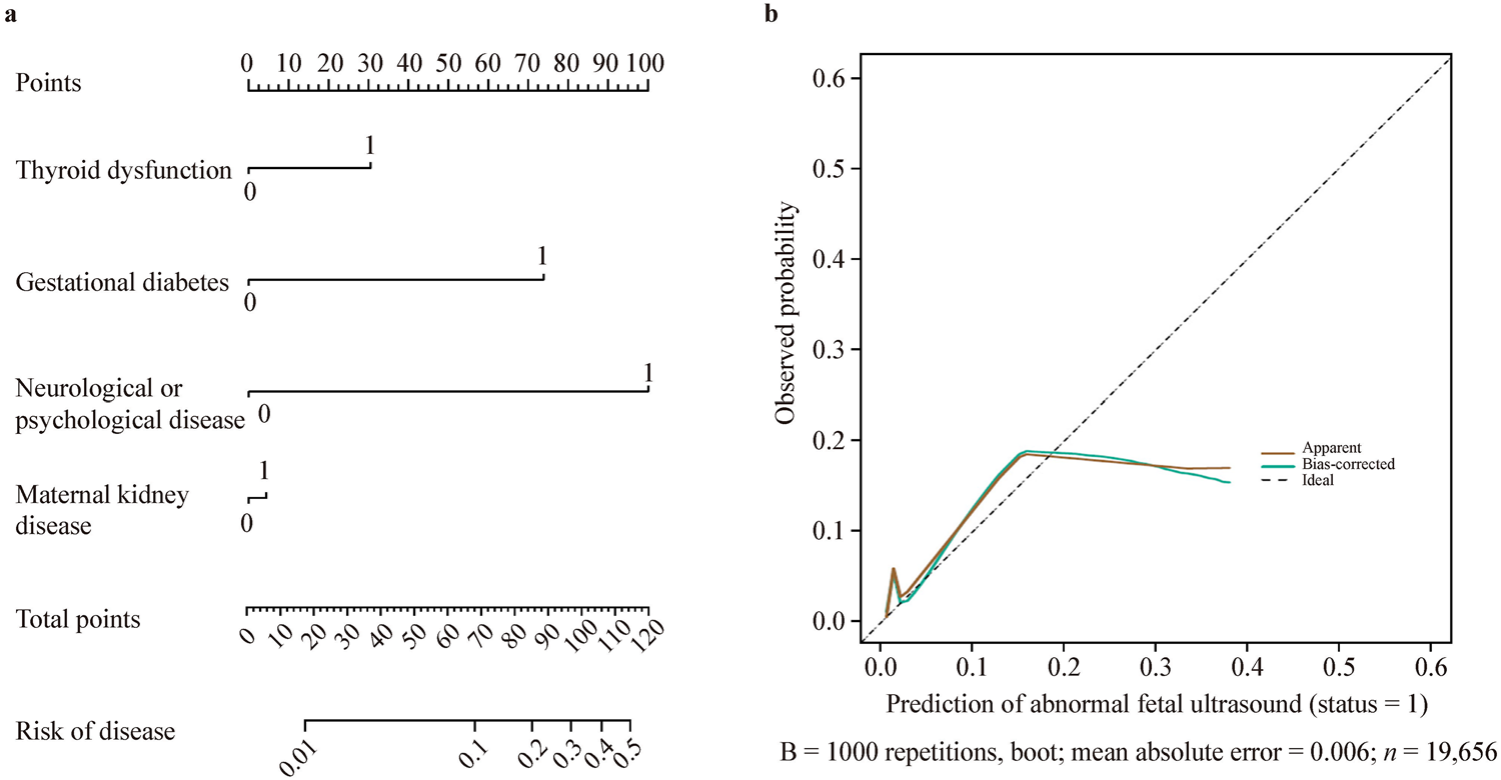
Prediction of abnormal findings by prenatal ultrasound screening for congenital anomalies of the kidney and urinary tract. **a** Nomogram predicting postnatal ultrasound screening result. To utilize the nomogram, an individual patient’s value was presented on each variable axis, and the vertical line was down upward to find the number of points received for each variable value. The sum of the variable values was presented on the total point axis, and a vertical line was drawn downward to the probabilities of abnormal screening outcome; **b** calibration curve of the predictive model. The X-axis showed the predicted abnormal screening results. The Y-axis showed the actual abnormal screening results. The vertical lines show the frequency distribution of the predicted positive ratio of screening result. The apparent calibration curve (dotted line) indicates the model performance in the original data, while the bias-corrected curve (solid line) represents the model performance after optimism correction using 1000 bootstrapped resamples. A perfect prediction would fall on the 45-degree (dashed) reference line

Based on the multivariate logistic regression analysis, a nomogram was constructed by assigning a weighted point to each independent risk factor on the point scale (Fig. 3a). A higher total point of all confounding factors refers to a higher possibility of suspicious CAKUT by antenatal ultrasound screening. The multivariate model was applied to the confounding factors (gestational diabetes, neuropsychiatric disease, thyroid dysfunction, and maternal kidney disease) named model 1. Discrimination and calibration, two basic characteristics of model validation, were tested to evaluate the performance of nomogram model 1. Model discrimination was assessed using the C-index which measures the ability to predict the outcomes. A higher C-index refers to a greater ability to discriminate the outcomes. The C-index of our nomogram model 1 was 0.732 (95% CI = 0.731–0.733), showing a great discrimination ability. The calibration plot revealed good predictive accuracy between the actual probability and predicted probability (Fig. 3b).

**Fig. 4:**
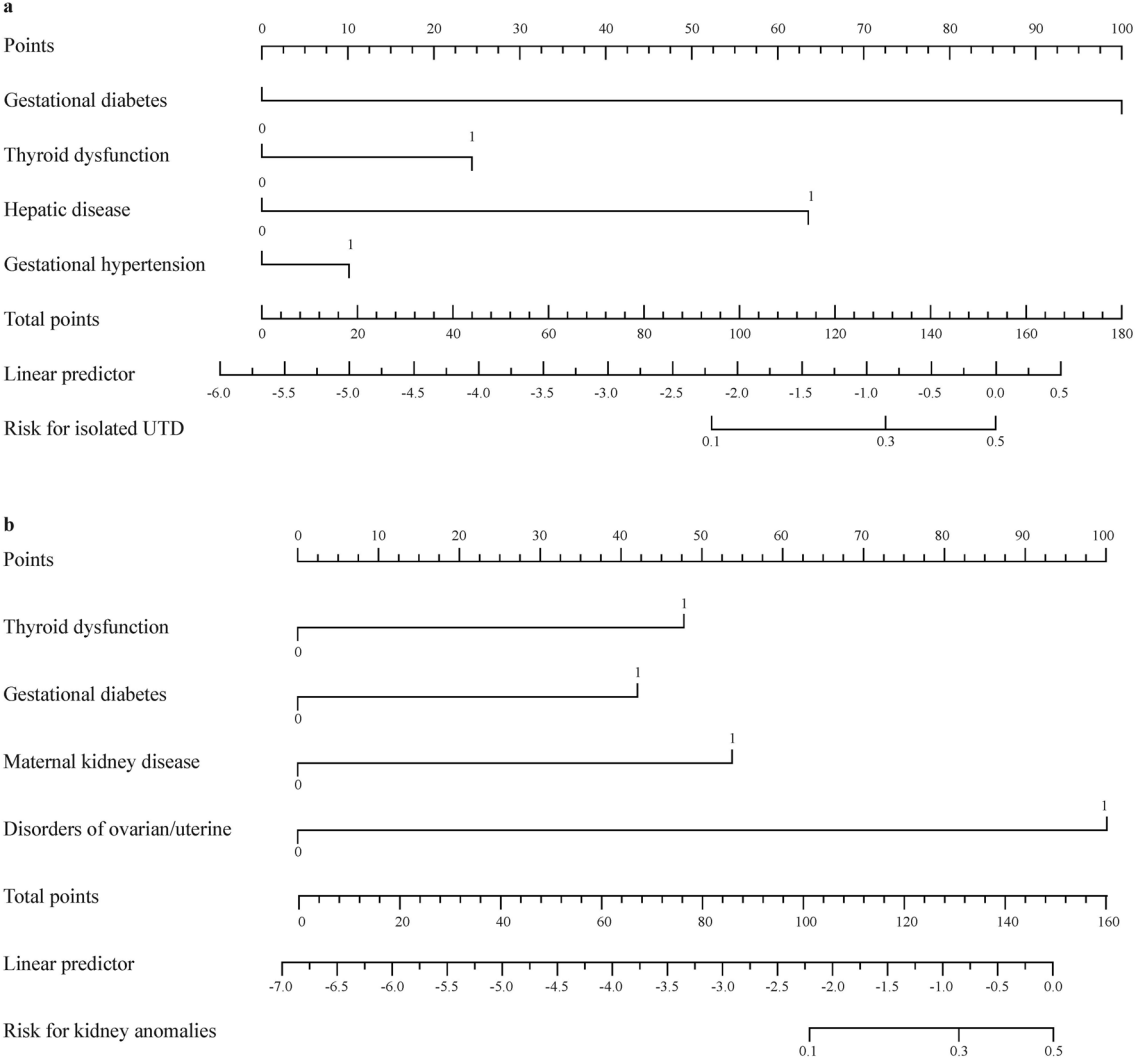
Nomograms for the prediction of different subtypes of congenital anomalies of the kidney and urinary tract. **a** Prediction for isolated urinary tract dilation (UTD); **b** prediction for kidney anomalies

Meanwhile, we constructed two nomograms that could be applied in different situations. A vertical line drawn from the scale value of each predictor down to the “score” scale provides the numerical score for that predictor. The sum of the predictors yields the “total score” which can be scaled to the final output probability of isolated UTD or antenatally detected kidney abnormalities. A prediction model for isolated UTD was developed utilizing four confounding factors, namely gestational diabetes, gestational hypertension, thyroid dysfunction, and hepatic disease (model 2, Fig. 4a). Similarly, a separate prediction model for KA was established based on four distinct confounding factors, namely thyroid dysfunction, gestational diabetes, ovarian/uterine disorders, and maternal kidney disease (model 3, Fig. 4b). For example, a mother with gestational diabetes and hypothyroidism was associated with a total score of points 125 points. The score indicated that this offspring had a 30% risk of isolated UTD detected by ultrasound antenatally as shown in Fig. 4a. Another example is a mother with gestational diabetes and maternal kidney disorders as shown in Fig. 4b. The score of 100 points indicated that this offspring had a 10% risk of KA detected by ultrasound antenatally.

## Discussion

This retrospective cohort study investigated the associations between various maternal diseases and CAKUT detected antenatally in offspring. Statistical analysis showed that gestational diabetes, thyroid dysfunction, neuropsychiatric disease, anemia, and ovarian and uterine disorders are associated with antenatally detected CAKUT. The associations showed differing patterns between patients with isolated UTD and KA. The risk of isolated UTD was increased in the fetuses of mothers with gestational diabetes, gestational hypertension, thyroid dysfunction, or hepatic disease. In contrast, the risk of KA was increased in the fetuses whose mothers had kidney disease, thyroid dysfunction, gestational diabetes, or ovarian/uterine disorders.

The antenatal detection ratio of CAKUT in the present study was 5.8 per 1000 pregnancies, which is within the lower range reported previously [8–10]. The prevalence of CAKUT in recent population studies was reported to range from 0.4% to 1.1% in term infants and 2.0% in preterm infants [5]. Variations between different reports may be associated with the criteria of CAKUT diagnosis, the database used for information retrieval, variations in sample size, and ethnic differences. From 2020 to 2022, the birth rate in China changed from 8.52‰ to 6.77‰ [11]. At the end of 2022, Tai’an had a permanent population of 1.25 million with a birth rate of 4.05 per 1000 population. Based on the estimation, the cohort of 19,656 pregnant women who participated in this study represented a significant proportion of the pregnant population in Tai’an City from 2020 to 2022.

Antenatal UTD is one of the most common fetal anomalies detected during pregnancy [1, 12]. It is commonly observed that the majority of dilatations tend to resolve without intervention, which is referred to as physiological or transient dilatation [12]. Nevertheless, there are some instances where UTD may be the initial indication of an underlying urinary tract anomaly [13, 14]. In the present study, the antenatal detection rate of isolated UTD was found to be 4.5‰, which was comparable with previous reports [15, 16]. These results provided further consultations for postnatal follow-up among the offspring. A meta-analysis found that 12% of infants with mild dilatation, 45% with moderate dilatation, and 88% with severe fetal dilatation were postnatally diagnosed with CAKUT [17].

The KA detected antenatally in this study were predominantly characterized by the malformations of hypo/dysplasia, ectopia, and lower urinary tract obstruction, with a prevalence of 1.3‰. Individuals with reduced nephron endowment, which is a manifestation of CAKUT, can be identified as having chronic kidney disease (CKD) in adulthood. We provided perinatal close follow-up for pregnant mothers and fetuses [1]. Postnatal monitoring of kidney function, electrolyte level, and ultrasound scanning were prescribed for the newborns. KA are high-risk factors associated with acute kidney injury in neonates [18].

In our study, maternal gestational diabetes was associated with isolated UTD as well as with KA in the fetus. Several epidemiological studies have provided evidence of a correlation between maternal diabetes and congenital malformations overall, as well as kidney malformations [8, 19]. The correlation may be attributed to maternal hyperglycemia. In animal models, maternal hyperglycemia has been shown to have a negative impact on fetal kidney development, including ureteric bud branching morphogenesis and nephron endowment [20, 21]. The significance of hyperglycemia during the early stage of pregnancy, when a process of active nephrogenesis occurs, may be supported by the significantly high likelihood of KA (adjusted OR of 6.8 shown in our data) in fetuses of mothers with gestational diabetes. A recent study showed the association of hemoglobin A1c with complicated CAKUT with extrarenal phenotypes [8]. The levels of hemoglobin A1c during early pregnancy may be lower, even in cases of gestational diabetes, compared to the nonpregnant state [8]. In addition, exposure to such levels could be sufficient to increase the risk of adverse pregnancy outcomes [22].

The risk of isolated UTD or KA was also increased in the fetuses of mothers with thyroid dysfunction. The expected incidence of gestational hypothyroidism or hyperthyroidism ranges from 0.3% to 11%, depending on the definition and geographical area [23]. We have presented a prevalence of 2.2% for maternal thyroid dysfunction. As is well known about the association of maternal hypothyroidism and preterm birth [24], the findings of preterm birth associated with CAKUT [5] may be due to maternal thyroid dysfunction. A cohort from another city in China documented the associations between maternal hypothyroidism and postnatal CAKUT in either preterm infants or term infants [6]. Further investigation should be conducted on the potential teratogenic effects of drugs which are contraindicated for pregnant women but might be used if the pregnancy was unplanned or suboptimally managed.

The nomogram presents a novel approach to providing personalized predictive information, based on risk factors that have demonstrated significant differences in multivariate analysis. Currently, in the field of CAKUT, most studies have utilized logistic regression to identify risk factors, yet a predictive model has yet to be established. Here, we built a nomogram for predicting antenatal CAKUT using a new statistical method, which can extract as much information as possible to provide the most accurate predictions from maternal factors. The nomogram prediction model could be a useful tool for estimating the maternal risks of CAKUT in offspring. It would provide an avenue for targeted screening and close follow-up of individuals with a high risk of CAKUT.

Several limitations of our study should be noted. Firstly, the risk factors associated with CAKUT during the fetal stage may be missed in situations such as miscarriage, termination of pregnancy, stillbirth, or death of fetuses prenatally diagnosed with KA. Secondly, since the diagnoses of maternal diseases were partially obtained from self-reported questionnaires, some diagnoses may have been overlooked, biased, or inconsistent, especially in cases of preexisting chronic hypertension, thyroid dysfunction, or diabetes. Furthermore, the diagnosis of maternal kidney disease was not specified (e.g., CAKUT, CKD, chronic nephritis, and proteinuria were possible for “kidney disease”). There was recall bias due to the reliance on self-reported maternal health conditions. In addition, maternal health conditions such as neurological disease and anemia might be underreported. Collection of medication history would help with the identification of maternal diseases. Thirdly, we were able to examine the association of maternal diseases with UTD and KA detected antenatally. It has been demonstrated that children with any degree of antenatal hydronephrosis are at greater risk of postnatal pathology compared to the normal population [12, 17]. Postnatal follow-up for the offspring in another ongoing study would provide further knowledge on the associations of the risk factors and postnatal diagnosis of CAKUT. Finally, the risk of KA was increased in fetuses of mothers with kidney disease in this study. This finding is significant because 10%–15% of cases with CAKUT show familial aggregation [1]. In the absence of genetic testing, it is not possible to definitively ascertain the veracity of consolidating all instances of isolated CAKUT.

In conclusion, maternal diseases, especially gestational diabetes mellitus and thyroid dysfunction, are highly associated with CAKUT in offspring. Various subtypes of CAKUT were associated with different maternal diseases. Our results may facilitate the early identification of women at the highest risk of bearing children with CAKUT and the provision of more specific preconception counseling and subsequent follow-up for newborns.

## Data Availability

The datasets for this article are not publicly available. Requests to access the datasets should be directed to the corresponding authors.
